# Accumulation of Toxic Arsenic by Cherry Radish Tuber (*Raphanus sativus* var. *sativus* Pers.) and Its Physiological, Metabolic and Anatomical Stress Responses

**DOI:** 10.3390/plants12061257

**Published:** 2023-03-10

**Authors:** Daniela Pavlíková, Milan Pavlík, Veronika Zemanová, Milan Novák, Petr Doležal, Petre I. Dobrev, Václav Motyka, Kamil Kraus

**Affiliations:** 1 Department of Agro-Environmental Chemistry and Plant Nutrition, Faculty of Agrobiology, Food and Natural Resources, Czech University of Life Sciences Prague, Kamýcká 129, 165 00 Prague, Czech Republic; 2 Isotope Laboratory, Institute of Experimental Botany of the Czech Academy of Sciences, Vídeňská 1083, 142 20 Prague, Czech Republic; 3 Laboratory of Hormonal Regulations in Plants, Institute of Experimental Botany of the Czech Academy of Sciences, Rozvojová 263, 165 02 Prague, Czech Republic; 4 Department of Botany and Plant Physiology, Faculty of Agrobiology, Food and Natural Resources, Czech University of Life Sciences Prague, Kamýcká 129, 165 00 Prague, Czech Republic

**Keywords:** metalloid, methionine, stress metabolism, vegetable, vitamin C

## Abstract

In a pot experiment, cherry radish (*Raphanus sativus* var. *sativus* Pers. ‘Viola’) was cultivated under two levels of As soil contamination—20 and 100 mg/kg. The increasing As content in tubers with increasing soil contamination led to changes in free amino acids (AAs) and phytohormone metabolism and antioxidative metabolites. Changes were mainly observed under conditions of high As contamination (As100). The content of indole-3-acetic acid in tubers varied under different levels of As stress, but As100 contamination led to an increase in its bacterial precursor indole-3-acetamide. A decrease in *cis*-zeatin-9-riboside-5′-monophosphate content and an increase in jasmonic acid content were found in this treatment. The free AA content in tubers was also reduced. The main free AAs were determined to be transport AAs (glutamate—Glu, aspartate, glutamine—Gln, asparagine) with the main portion being Gln. The Glu/Gln ratio—a significant indicator of primary N assimilation in plants—decreased under the As100 treatment condition. A decrease in antioxidative metabolite content—namely that of ascorbic acid and anthocyanins—was observed in this experiment. A decline in anthocyanin content is related to a decrease in aromatic AA content which is crucial for secondary metabolite production. The changes in tubers caused by As contamination were reflected in anatomical changes in the radish tubers and roots.

## 1. Introduction

Toxic arsenic (As) is one of the elements that are commonly present in the environment. It is the twentieth most abundant element in the Earth’s crust [1]. In small quantities, it is practically always present in animal and plant tissues. Mining and smelting of arsenic-bearing minerals are the main anthropogenic sources of As contamination in the environment. In soils, its inorganic forms arsenate (As^V^) and arsenite (As^III^) predominate. Arsenate is the dominant species in conditions of oxidative soil; As^III^ predominates in reducing conditions. Arsenite has a more toxic effect on plants than As^V^. Increased As content in soils used for farming and gardening is a potential risk for human health and transfer through the food chain [2].

Arsenic uptake and translocation from roots to shoots, including the reduction rate of As^V^ to As^III^ and the fate of As, vary among different plant species [3]. Paltseva et al. [2] analyzed statistically significant differences in As contents of vegetables and confirmed the highest As content in the edible part of radish, followed by lettuce, carrots and then tomatoes. This finding is consistent with the results of [4,5]. According to these authors, vegetable type is a strong determinant of toxic element concentration.

Both forms of inorganic As are reported to induce oxidative stress. It has been published that the generation of reactive oxygen species (ROS) in plants is linked to the reduction of As^V^ to As^III^. Enhanced ROS production leads to the destruction of photosynthesis apparatus, DNA, amino acids, proteins and membrane lipids [6,7,8]. The plant defense system increases the activities of antioxidant enzymes as protection from oxidative damage caused by ROS such as superoxide radical (O^2−^), hydroxyl radical (OH) and hydrogen peroxide (H_2_O_2_). The basic defense against a ROS imbalance is the ascorbate–glutathione cycle containing enzymatic antioxidants superoxide dismutase, ascorbate peroxidase, catalase, glutathione peroxidase, glutathione reductase and two non-enzymatic compounds ascorbic acid and glutathione. The other antioxidative metabolites such as flavonoids, tocopherols and carotenoids have a supporting role [3,9].

The binding of As by phytochelatins accumulates most of the As in the roots and a much smaller amount of As is translocated into the aboveground part of the plant [10]. According to [11], carrot roots had higher As concentrations than lettuce leaves because the direct soil contact of carrots leads to higher concentrations compared to the lettuce which must translocate As from roots to shoots. Experiments by [12,13] did not confirm this speculation. According to these authors, the formation of the As complex with phytochelatins restricts the transport of As from the roots to the aboveground parts of plants.

Primary As detoxification in plant cells relies on the reduction of As^V^ to As^III^ and on the formation of As^III^–glutathione or As^III^–phytochelatin complexes, which can be transported to the vacuole [3]. High As concentrations induce a specific plant stress metabolism, determine transport and elimination and inhibit metabolic processes, such as changes in phytohormone and amino acid contents, photosynthetic processes, epigenetic changes, etc. [14,15]. 

In the present study, edible parts of cherry radish tubers were used for the investigation of anatomical and physiological changes in plants grown under As stress. We assume that due to low and high levels of As soil contamination and As accumulation in radish tubers, changes in free amino acids (AAs) and phytohormone metabolism and antioxidant activities occur.

## 2. Results

### 2.1. Arsenic Content and Effect on Biomass of Cherry Radish

Cherry radish accumulated As mainly in its roots and tubers (Table 1). Root contents of both As treatments were one order higher than tuber contents. Root and tuber contents of As increased in an As dose-dependent manner as was confirmed by the correlation (r = 0.97 and 0.99 (*p* ˂ 0.001), respectively). However, for roots, the increase was significant only for the As100 treatment. In the leaves of cherry radish, the As content was under the detection limit of the chosen method (ICP-OES), except for those that underwent As100 treatment (Table 1).

The influence of As on the growth of radish was not statistically significant. Root growth declined and was in the range of 0.02 to 0.01 g of dry biomass per plant. The yield of dry biomass for tubers was in the range of 0.36 to 0.19 g per plant and that of dry biomass for leaves was in the range of 0.62 to 0.20 g per plant.

### 2.2. Phosphorus and Sulphur Content under Arsenic Exposure

**Figure 1:**
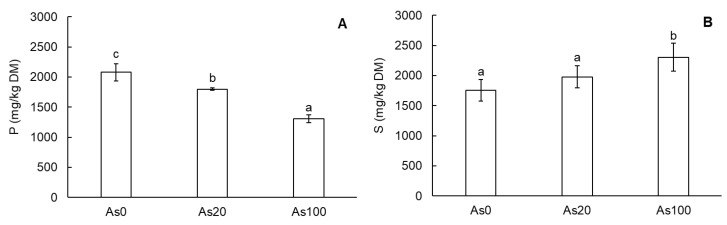
Content of phosphorus (**A**) and sulfur (**B**) in tubers of cherry radish. Values represent the mean ± SD. Data with the same letter are not significantly different. Different letters indicate significant differences (*p* ˂ 0.05) among treatments according to ANOVA with Tukey’s post-hoc test. Abbreviations: As0—0 mg As/kg soil (control); As20—20 mg As/kg soil; As100—100 mg As/kg soil; DM—dry matter.

In the tubers of cherry radish, both selected elements—phosphorus (P) and sulfur (S), both important for the homeostasis of plant metabolism—were affected by As contamination (Figure 1A,B). Phosphorus content in tubers decreased in an As dose-dependent manner as was confirmed by the correlation with As in the soil (r = −0.95, *p* ˂ 0.001) as well as As content in tubers (r = −0.94, *p* ˂ 0.001). As treatments As20 and As100 decreased P content by 13.5% and 37%, respectively (Figure 1A). Further, S content in tubers was correlated with the As concentration in the soil (r = 0.75, *p* ˂ 0.001) and As content in tubers (r = 0.74, *p* ˂ 0.001). However, in contrast to P content, this element was enhanced by As contamination, especially by the As100 treatment which increased S by 31% compared to the control (Figure 1B).

### 2.3. Phytohormone Content under Arsenic Exposure

**Figure 2:**
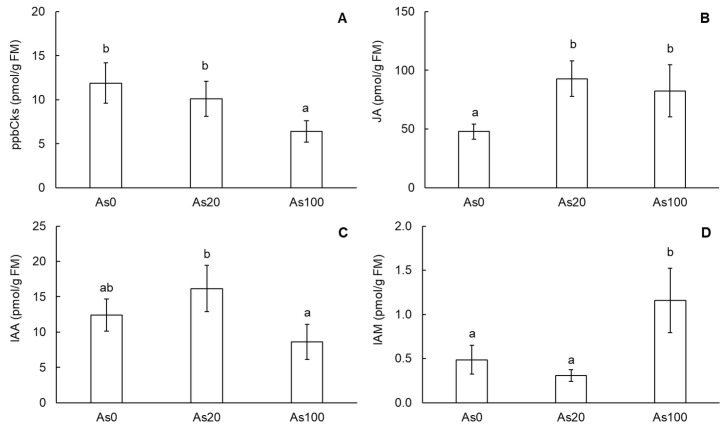
Content of primary products of cytokinins biosynthesis (**A**), jasmonic acid (**B**), indole-3-acetic acid (**C**) and indole-3-acetamide (**D**) in tubers of cherry radish. Values represent the mean ± SD. Data with the same letter are not significantly different. Different letters indicate significant differences (*p* ˂ 0.05) among treatments according to ANOVA with Tukey’s post-hoc test. Abbreviations: As0—0 mg As/kg soil (control); As20—20 mg As/kg soil; As100—100 mg As/kg soil; FM—fresh matter.

To evaluate the response of cherry radish to As contamination, the content of phytohormones was determined in tubers. Among phytohormones, a significant change in content by soil As concentration was determined for the group of primary products of cytokinins biosynthesis (ppbCKs) as well as jasmonic acid (JA), indole-3-acetic acid (IAA) and its bacterial precursor indole-3-acetamide (IAM) in cherry radish tubers (Figure 2A–D). The ppbCKs group, which includes inactive and very low-activity CK nucleotides, was decreased under As100 treatment conditions (by 46%, Figure 2A) and correlated with the As dose in the soil (r = −0.82, *p* ˂ 0.001) as well as the As content in tubers (r = −0.80, *p* ˂ 0.001). The most abundant ppbCK phytohormone was cis-zeatin-9-riboside-5′-monophosphate (*cis*ZRMP). *cis*ZRMP content represented 61.3–63.9% of ppbCKs and showed the same trend as this group in response to As treatment. In tubers of cherry radish, *cis*ZRMP was decreased by the As100 treatment (by 48.5%) compared to the control and correlated with the As dose in the soil (r = −0.81, *p* ˂ 0.001) as well as the As content in tubers (r = −0.79, *p* ˂ 0.001). 

In contrast to ppbCKs, JA content in cherry radish tubers was increased by both As treatments (Figure 2B). Compared to the control, As20 and As100 treatments increased the JA content by 94% and 73%, respectively. However, a correlation between JA content and As concentration in the soil as well as the As content in tubers was not observed. The last phytohormones that showed a significant change in content in cherry radish tubers in response to As contamination were auxins, more precisely IAA and IAM (Figure 2C,D). The IAA, an active form of auxins, showed significantly different content between As treatments—ca. two-fold lower content in tubers under As100 treatment conditions compared to tubers undergoing As20 treatment (Figure 2C). However, compared to the control, the change was not statistically significant. Despite this, IAA content was correlated with soil As concentration (r = −0.62, *p* ˂ 0.01) and As content in tubers (r = −0.70, *p* ˂ 0.001). The reduced conversion of IAM to IAA in the tubers of cherry radish was suggested based on the change in IAM content due to high levels of As contamination. The As100 treatment increased the IAM content by 138% compared to the control (Figure 2D). Content of this phytohormone correlated with soil As concentration (r = 0.82, *p* ˂ 0.001) and As content in tubers (r = 0.86, *p* ˂ 0.001).

### 2.4. Free Amino Acid Content under Arsenic Exposure

In cherry radish tubers, a change in free AA metabolism due to low and high levels of As contamination was observed. The contents of 21 free AAs and amides were determined and presented as the total content of free AAs (Table 2). Compared to the control, low levels of As contamination (As20 treatment) increased the total content of free AAs by 16.7% while high levels of As contamination (As100 treatment) decreased the total content of free AAs by 17.8%. This content correlated with soil As concentration (r = −0.62, *p* ˂ 0.001) and As content in tubers (r = −0.69, *p* ˂ 0.001). 

Among free AAs, the highest abundance was determined for the group of transport AAs that includes glutamate (Glu), aspartate (Asp) and their amides glutamine (Gln) and asparagine (Asn). The content of transport AAs represented 56.3–73.6% of total free AA content (Table 2). Compared to the control, As20 and As100 treatments increased the content of transport AAs by 16.1% and 6.8%, respectively. However, the effect was significant only for low levels of As contamination. A correlation between this group of free AAs and soil As concentration as well as As content in tubers was not observed.

Among treatments, Gln was the most abundant free AA in tubers of cherry radish (30.4—49.9% of total content). Compared to the control, As20 and As100 treatments increased Gln content by 15.9% and 33.8%, respectively. However, the effect was significant only for high levels of As contamination (Table 2). Despite this, Gln content was correlated with soil As concentration (r = −0.65, *p* ˂ 0.01) and As content in tubers (r = −0.61, *p* ˂ 0.01). The ratio of Glu and Gln was used as an indicator of changes in AA regulation. The effect of As20 treatment on this ratio was negligible (Table 2) while As100 treatment decreased the ratio by 44.8% compared to the control and suggested a toxic effect associated with this high level of As contamination.

In the tubers of cherry radish, we also determined a group of three aromatic AAs—free phenylalanine (Phe), free tryptophan (Trp) and free tyrosine (Tyr)—that play a role in the stress response of plants. The content of aromatic AAs represented 0.6—1.5% of the total free AA content (Table 2). Compared to the control, As20 and As100 treatments decreased the content of aromatic AAs by 43.2% and 66.1%, respectively. This group of free AAs correlated with soil As concentration (r = −0.70, *p* ˂ 0.001) and As content in tubers (r = −0.63, *p* ˂ 0.01). Furthermore, toxicity associated with high levels of As contamination (As100 treatment) was suggested by the results of methionine (Met) content. Content of Met represented only 0.06—0.2% of total free AA content (Table 2); however, As100 treatment increased Met content by 110.1% compared to the control. Additionally, a correlation confirmed the relationship between Met and soil As concentration (r = 0.74, *p* ˂ 0.001) as well as As content in tubers (r = 0.79, *p* ˂ 0.001).

### 2.5. Total Anthocyanin Content and Ascorbic Acid under Arsenic Exposure

**Figure 3:**
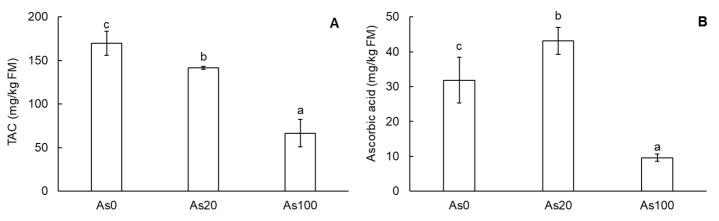
Total anthocyanin content (**A**) and ascorbic acid (**B**) in tubers of cherry radish. Values represent the mean ± SD. Data with the same letter are not significantly different. Different letters indicate significant differences (*p* ˂ 0.05) among treatments according to ANOVA with Tukey’s post-hoc test. Abbreviations: As0—0 mg As/kg soil (control); As20—20 mg As/kg soil; As100—100 mg As/kg soil; TAC—total anthocyanin content (mg/kg FM); FM—fresh matter.

The results of antioxidative metabolites—total anthocyanin content (TAC) and ascorbic acid content—are presented in Figure 3A,B. In cherry radish tubers, TAC decreased in an As dose-dependent manner as was confirmed by the correlation with As in the soil (r = −0.97, *p* ˂ 0.001) as well as As content in tubers (r = −0.95, *p* ˂ 0.001). Compared to the control, TAC in the tubers undergoing As20 and As100 treatments decreased by 17% and 61%, respectively (Figure 3A). In the case of ascorbic acid, the content in cherry radish tubers was affected by As contamination with an opposite trend. Compared to the control, the As20 treatment increased ascorbic acid content by 35%, while the As100 treatment decreased ascorbic acid content by 70% (Figure 3B). Content of this non-enzymatic antioxidant was correlated with soil As dose concentration (r = −0.83, *p* ˂ 0.001) as well as As content in tubers (r = −0.90, *p* ˂ 0.001).

### 2.6. Anatomy of Cherry Radish under Arsenic Exposure

**Figure 4:**
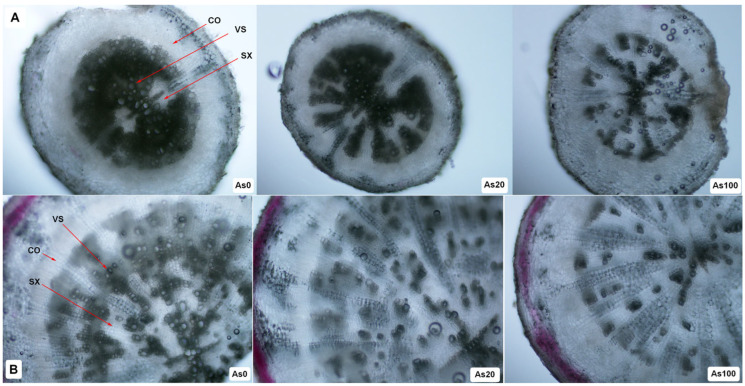
Cross section through root (**A**, 100× magnification) and tuber (**B**, 200× magnification) of cherry radish. Abbreviations: As0—0 mg As/kg soil; As20—20 mg As/kg soil; As100—100 mg As/kg soil; CO—cortex; SX—secondary xylem; VS—vessels.

Although the influence of As on the growth of radish was not statistically significant, cross-sectional analysis of roots and tubers showed changes in the anatomical structure due to As exposure (Figure 4A,B). Increasing the As dose in the soil affected the anatomical characteristics of cherry radish roots and tubers, especially the differentiation of the secondary xylem. Roots exhibited a higher differentiation of the secondary xylem than tubers. Compared to the control, the roots of plants that underwent As treatments showed smaller-diameter vessels in radially arranged groups (Figure 4A). Furthermore, the tubers of plants that underwent As treatments showed a smaller diameter of vessels, thinning of the anthocyanidin layer as well as a reduction in pigment color under the periderm (Figure 4B).

## 3. Discussion

The growth of radish plants declined with increasing As content in the soil of a pot experiment in contrast to the control. Our results show significantly increasing As content in tubers with increasing soil contamination (Table 1). The same finding was described by [2] who confirmed high As content in the edible part of radishes. Further, Smith et al. [13] determined As accumulation in tubers. Arsenic was present as As^V^ and comprised 77–92% of the total As content. In general, As contamination affected the uptake and content of elements in plants. Its inorganic form—As^V^, a major form in aerobic soil—is, structurally, a chemical analogue of phosphate and enters plant roots via phosphate (Pi) transporters [16]; therefore, the decline in P content in radish tubers was associated with increasing As content as our results indicated (Figure 1A). 

A decline in P content in plants negatively affects the metabolism of phosphor proteins—namely stress signal molecules [17], lipids contained in membranes [18], nucleic acids and *cis*ZRMP, which is a primary product of CKs biosynthesis. Our results show a decrease in *cis*ZRMP content by 48.5% in the As100 treatment in contrast to the control. The decrease in the contents of the precursors to bioactive CKs (such as *cis*ZRMP) pointed to plant senescence [19]. 

In root systems, sufficient levels of IAA, the auxin growth hormone, are required for the formation, development and maintenance of the roots [20]. Our results show that IAA content in cherry radish tubers was varied under As stress, but strong As contamination led to an increase in its bacterial precursor—IAM (Figure 2C,D). The increase in IAM content in the As100 treatment resulted in a reduced conversion of IAM to IAA. The disturbed dynamic balance between IAA and IAM in cherry radish tubers could be caused by a disturbance in the activity of enzymes responsible for the conversion of Trp to IAA via IAM [21]. 

Jasmonic acid has a protective role in regulating plant stress responses but is also active in root growth and senescence. Our results indicated a significant increase in JA content in cherry radish tubers under As treatment in contrast with the control (Figure 2B). Increased JA synthesis in plants grown under As stress promotes the expression of some signaling and stress-responsive genes. Genes for this synthesis can be upregulated both in roots and shoots. It suggests that JA signaling may play a role in plants to cope with As stress [22]. 

Our results show significantly increased S content in cherry radish tubers grown in the most contaminated soil (Figure 1B). Sulfur is known for its role in AAs cysteine and methionine, phytohormones, toxic element chelators, etc., and, therefore, it induced As plant stress tolerance [23]. According to Shi et al. [24], S in plants reduced the root-to-shoot As translocation because of induced phytochelatin synthesis and As transfer into vacuoles.

Amino acid metabolism has a central role in abiotic oxidative stress in adapted plants. The effect of As on the plant metabolism of AAs has been studied in many plant species [19,25,26,27]. The content of AAs can be negatively affected by stress and some free AAs play a protective role in stress tolerance in different ways such as intracellular pH regulation, substrates for glutathione formation, etc. [28,29]. As soil contamination led to an increase in free AA content in the roots and aboveground biomass of tomato plants in contrast to a control [27]. Similar results were published for *Spinacia oleracea*, but an opposite trend was confirmed for As hyperaccumulator *Pteris cretica* [14]. These findings for tomato and spinach plants are consistent only with our results for cherry radish under conditions of As20 treatment. As a consequence of high As exposure (As100 treatment), the AA content in tubers decreased (Table 2). Among free AAs in *Spinacia oleracea*, the highest content was determined for transport AAs [14,25]. The main AAs in the roots of tomato [27], similar to the cherry radish tubers in our experiment, were also determined to be transport AAs (Glu and Asp and their storage amides—Gln and Asn). Plants exposed to oxidative stress need Glu for glutathione biosynthesis because this metabolite is essential in the glutathione–ascorbate cycle. Glutamine, an amide involved in the synthesis of other AAs, formed the main portion of free AAs in our experiment (Table 2). The increase in its content in plants grown under As stress may be due to changes in the activities of glutamate synthase and glutamine synthetase enzymes [27,30]. The Glu/Gln ratio in cherry radish tubers decreased with the highest content of As in the soil (Table 2). Similar results were published for *P. cretica* and *P. straminea* and the Glu/Gln ratio is presented as a significant indicator of primary N assimilation [19,31]. Methionine (sulfur AA) is an AA whose content in cherry radish tubers increased due to As stress. The role of Met in overcoming As toxicity is associated with the transmethylation cycle, which is essential for the biosynthesis of antioxidant secondary metabolites [32] and DNA methylation/demethylation. DNA demethylation allows the reactivation of silenced genes related to the stress metabolism of plants [33]. 

Plant senescence is related to oxidative stress caused by As contamination [3,34]. Plants under oxidative stress activate the antioxidative enzymatic system [35]. The contents of non-enzymatic antioxidants such as ascorbic acid, a significant antioxidative metabolite and part of the ascorbate–glutathione cycle, were increased under low As stress, but were decreased under high levels of As contamination and chronic stress [36,37]. This finding was confirmed by our results. While increased content of ascorbic acid was determined for the As20 treatment, the opposite result was found for the As100 treatment (Figure 3B). TAC, another antioxidative metabolite, decreased under oxidative stress in our experiment (Figure 3A). As in the cherry radish tubers in our results, in hypocotyls of radish, a decrease in TAC was found [38]. Decline in TAC is related to a decrease in aromatic AA content which is crucial for the production of secondary metabolites such as anthocyanins [29].

The above-mentioned changes in measured physiological and metabolic indicators caused by the toxic effects of As accumulation in plants [33] were reflected in anatomical changes in cherry radish tubers and roots (Figure 4). Marconi et al. [39] analyzed structural changes in radish tubers grown in soils contaminated by As. Consistent with our results, they confirmed a progressive collapse of the tubers’ structure with increasing As contamination. Black spots over the whole hypocotyl were observed, and changes in the thickness and structural details of the outermost cell layer were determined. According to these authors, a physiological response of the hypocotyl to As presence was found. The aim of this response was the creation of a barrier for As accumulation. The microscopic study of [40] confirmed morpho-anatomical changes in wheat roots grown under toxic metal stress. Mainly, the thickness of the root epidermis was decreased and ground tissue cells in the cortex changed their size and structure. Changes in the root structure of the As hyperaccumulator *P. cretica* were recorded by cross-sectional analysis through adventitious roots [33]. The roots of As-treated plants showed thinning of the sclerenchymatous inner cortex and a reduction in average tracheid metaxylem in the vascular cylinder, compared to controls.

## 4. Materials and Methods

### 4.1. Pot Experiment

A pot experiment was conducted in a randomized design with four replications of each treatment. The plastic pot was filled with 2.5 kg of soil from a non-polluted area (Table 3). Each kilogram of soil was mixed with 0.5 g N, 0.16 g P and 0.4 g K (applied as NH_4_NO_3_ and K_2_HPO_4_ solutions). The experimental design included the following treatments: As0—control without added As dose; As20—20 mg As/kg of soil; and As100—100 mg As/kg of soil. Arsenic was added in the form of Na_2_HAsO_4_ solution and mixed into the soil. The maturation period of the spiked soil was three months. Two As doses were chosen to represent low and high soil contamination.

Seeds of cherry radish (*Raphanus sativus* var. *sativus* Pers. ‘Viola’) from the company Nohel Garden a.s. were purchased from a store (in the Czech Republic) and were sown directly into the soil in pots (15 seeds per pot). Thinning was performed after two true leaves developed, keeping six seedlings in each pot. The pot experiment was carried out under greenhouse conditions (natural light conditions; temperature was 20–23 °C during the day and 15–18 °C at night; relative humidity ~60%). Cherry radishes were harvested after 50 days of growth and divided into roots, tubers and leaves. Each part was washed with distilled water, blotted dry with filter paper and weighed. Samples were partitioned for further analysis. One portion was immediately frozen in liquid nitrogen and stored at –80 °C until analysis of metabolites, while the other portion was oven-dried to a constant weight (three days at 40 °C) and homogenized for element analysis.

### 4.2. Microscopic Observations

To inspect structural symptoms of As toxicity, cross sections through a root (approximately 5 mm from root tip) and tuber (the part of the hypocotyl–root junction) were performed using a Nikon E 200 microscope equipped with a DS camera head and the NIS-Elements application (Nikon Instruments, Inc., Melville, NY, USA).

### 4.3. Determination of Elements

Arsenic and other elements’ contents were determined by an Agilent 720 inductively coupled plasma optical emission spectrometer (ICP-OES; Agilent Technologies Inc., Santa Clara, CA, USA) after low-pressure microwave digestion as previously described [19].

### 4.4. Determination of Phytohormones

Phytohormones in tubers were extracted as previously described [41]. Analysis of phytohormones was performed with a LC/MS system consisting of a UHPLC 1290 Infinity II (Agilent Technologies Inc., Santa Clara, CA, USA) coupled with a 6495 Triple Quadrupole Mass Spectrometer (Agilent Technologies Inc., Santa Clara, CA, USA), operating in MRM mode, with quantification determined by the isotope dilution method. Data acquisition and processing were performed with Mass Hunter software B.08 (Agilent Technologies Inc., Santa Clara, CA, USA).

### 4.5. Determination of Free Amino Acids

Free amino acids (AAs) in tubers were extracted, derivatized and determined as previously described [42]. Extracts were derivatized using an EZ:faast kit (Phenomenex, Torrance, CA, USA) following the manufacturer’s instructions. The prepared samples were analyzed on a Hewlett Packard 6890N/5975 MSD gas chromatography–mass spectrometry system (GC-MS; Agilent Technologies Inc., USA) with a ZB-AAA 10 m × 0.25 mm AA analysis GC column. The oven temperature program and MS conditions were the same as previously described [43]. A chromatogram of the free AA standard is presented in Figure S1. 

### 4.6. Determination of Ascorbic Acid

Ascorbic acid in tubers was determined as previously described [44]. Extraction and analysis were performed using an Ascorbic Acid Assay Kit II (Sigma-Aldrich, St. Louis, MI, USA) and a Tecan Infinite^®^ M200 (Tecan Austria GmbH, Grödig, Austria).

### 4.7. Determination of Total Anthocyanin Content

Total anthocyanin content (TAC) in tubers was determined using the spectrophotometric method previously described [45] with some modifications. Ground fresh samples (1 g) were extracted with 25 mL of an 85:15 (*v*/*v*) mixture of methanol and 1.0 M HCl. The sample was ultrasonicated for 30 min., incubated in the dark with shaking for 24 h and filtered using 0.22 μm PTFE filters. Absorbance was measured on a UV–Vis spectrophotometer (Evolution 210, Thermo Scientific, Waltham, MA, USA) at 535 nm and the TAC was quantified according to the formula A × 288.21.

### 4.8. Statistical Analysis

Statistical processing of the results was carried out using Statistica 12.0 software (StatSoft, Tulsa, OK, USA). All data were checked for homogeneity of variance and normality (Levene and Shapiro–Wilk tests). The data are presented as the mean values and standard deviation (SD) for four replicates of each treatment. One-way ANOVA followed by a post-hoc comparison with Tukey’s test (*p* < 0.05) was used to identify statistically significant differences between treatments. Correlation analysis was performed using Pearson’s linear correlation (r; *p* < 0.05) to explore the relationship between different parameters.

## 5. Conclusions

In the present study, the anatomical and physiological changes in cherry radish tubers grown under As stress are established. Both low- and high-As soil contamination and increasing As content in radish tubers led to changes in free amino acids and phytohormone metabolism and antioxidative metabolites. The disturbed dynamic balance between IAA and IAM, the decrease in *cis*ZRMP content and the significant increase in JA content in tubers of radishes grown under the high levels of As contamination (100 mg As/kg of soil) were clearly demonstrated. It was confirmed that JA together with assimilated S and the sulfur AA methionine play a significant role in the defense against As stress. The important results obtained by means of analyses of free AAs can help to understand N metabolism in tubers grown under As exposure. The main AAs in tubers were determined to be the transport AAs (Glu and Asp and their storage amides—Gln and Asn). Glutamine formed the main portion of free AAs. The Glu/Gln ratio—a significant indicator of primary N assimilation in plants—decreased with high As contamination. The content of significant antioxidative metabolites—ascorbic acid and anthocyanins—decreased under oxidative stress in our experiment. The decline in TAC was related to a decrease in aromatic AA contents that are crucial for the production of secondary metabolites, such as anthocyanins. The above-mentioned changes caused by the toxic effects of As accumulation in plants were reflected in the anatomical changes in radish tubers and roots.

## Figures and Tables

**Table 1:** Content of As in dry biomass of cherry radish. Values represent the mean ± SD. Data with the same letter are not significantly different. Different letters indicate significant differences (*p* ˂ 0.05) among treatments according to ANOVA with Tukey’s post-hoc test. Abbreviations: As0—0 mg As/kg soil (control); As20—20 mg As/kg soil; As100—100 mg As/kg soil; nd—value is under the detection limit of ICP-OES (3 mg/L); DM—dry matter.

	As Content in Plant Parts (mg/kg DM)		
	leaves	tubers	root
As0	nd	3.6 ± 0.2 ^a^	12.3 ± 2.8 ^a^
As20	nd	4.7 ± 0.2 ^b^	25.5 ± 6.7 ^a^
As100	6.6 ± 0.9	31.1 ± 0.5 ^c^	353.0 ± 55.8 ^b^

**Table 2:** Content of free amino acids in tubers of cherry radish. Values represent the mean ± SD. Data with the same letter are not significantly different. Different letters indicate significant differences (*p* ˂ 0.05) among treatments according to ANOVA with Tukey’s post-hoc test. Abbreviations: As0—0 mg As/kg soil (control); As20—20 mg As/kg soil; As100—100 mg As/kg soil; AAs—amino acids; Gln—free glutamine; Glu—free glutamate; Met—free methionine; FM—fresh matter.

	As0	As20	As100
Total AAs content (μmol/kg FM)	3676.1 ± 443.2 ^b^	4291.6 ± 376.8 ^c^	3020.9 ± 376.9 ^a^
Transport AAs (μmol/kg FM)	2081.7 ± 210.7 ^a^	2416.9 ± 285.9 ^b^	2223.1 ± 289.7 ^ab^
Aromatic AAs (μmol/kg FM)	56.7 ± 18.4 ^b^	32.2 ± 9.8 ^a^	19.2 ± 1.2 ^a^
Gln (μmol/kg FM)	1127.2 ± 122.5 ^a^	1306.4 ± 189.8 ^ab^	1508.0 ± 214.4 ^b^
Met (μmol/kg FM)	2.7 ± 1.4 ^a^	2.6 ± 1.2 ^a^	5.7 ± 0.2 ^b^
Glu/Gln (−)	0.4 ± 0.03 ^a^	0.4 ± 0.06 ^a^	0.2 ± 0.02 ^b^

**Table 3:** Characteristics of experimental soil.

Location	Suchdol, Prague (50°8′8″ N, 14°22′43″ E)
Soil type and subtype	Haplic Chernozem
pH_KCl_ (−)	7.1
Cation exchange capacity (mmol_(+)_/kg)	258
Organic carbon (%)	1.83
Pseudo-total content of As (mg/kg)	16 ± 1.7
Water-soluble fraction of As (mg/kg)	0.10 ± 0.01

## Data Availability

The data presented in this study are available in the article.

## Supplementary Materials

The following are available online at https://www.mdpi.com/article/10.3390/plants12061257/s1, Figure S1: Chromatogram of free amino acid standard (level 200 nmol/mL).
