# Supplementary figures and images for: Accumulation of Toxic Arsenic by Cherry Radish Tuber (Raphanus sativus var. sativus Pers.) and Its Physiological, Metabolic and Anatomical Stress Responses
Source: Plants (Basel). 2023 Mar 10;12(6):1257. doi: 10.3390/plants12061257 (PMC10051939; doi:10.3390/plants12061257)

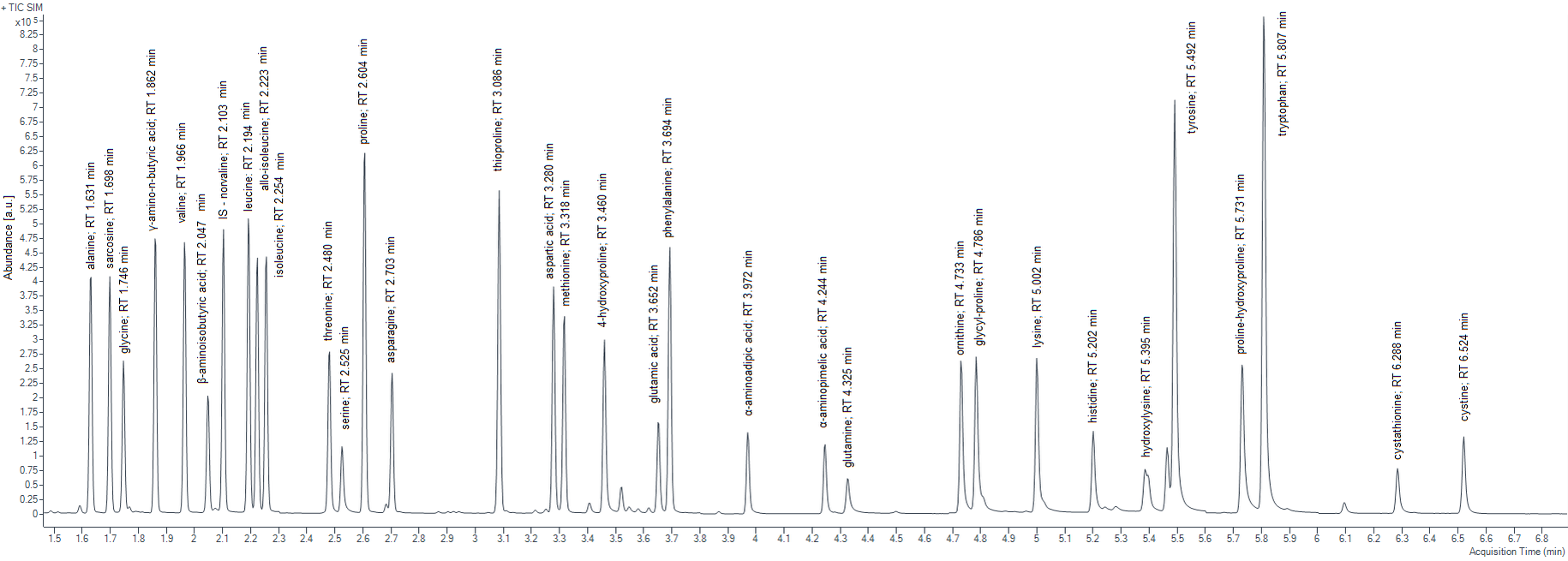

Supplement: Supplementary file 1 [file plants-12-01257-s001.zip › plants-2227978-supplementary.tif]
